# Correlation of cardiometabolic index and sarcopenia with cardiometabolic multimorbidity in middle-aged and older adult: a prospective study

**DOI:** 10.3389/fendo.2024.1387374

**Published:** 2024-05-28

**Authors:** Ling He, Chuyang Lin, Yansong Tu, Yazhi Yang, Ming Lin, Huaijun Tu, Jian Li

**Affiliations:** ^1^ The Department of Geriatrics, The Second Affiliated Hospital of Nanchang University, Nanchang, Jiangxi, China; ^2^ The Department of Clinical Research Center, The Second Affiliated Hospital of Nanchang University, Nanchang, Jiangxi, China; ^3^ The Department of Biochemistry and Pharmacology, The University of Melbourne, Parkville, VIC, Australia; ^4^ The Second Affiliated Hospital, Jiangxi Medical College, Nanchang University, Nanchang, Jiangxi, China

**Keywords:** CHARLS, cardiometabolic multimorbidity, cardiac metabolic index, visceral obesity, sarcopenia

## Abstract

**Background:**

Research has demonstrated that sarcopenia and visceral obesity are significant risk factors for chronic disease in middle-aged and older adults. However, the relationship between sarcopenia, the cardiac metabolic index (CMI), a novel measure of visceral obesity, and cardiometabolic multimorbidity (CMM) remains unclear. In this study, data from the China Longitudinal Study of Health and Retirement (CHARLS) were analyzed to investigate the association between sarcopenia and CMI with CMM in the middle-aged and older adult population.

**Methods:**

The study included 4,959 participants aged 45 and over. Sarcopenia was defined using the criteria of the Asian Sarcopenia Working Group 2019. CMM is defined as having two or more of the following conditions: physician-diagnosed heart disease, diabetes, stroke, and/or hypertension. CMI was calculated using the formula: CMI = (TG/HDL-C) × WHtR. To explore the association between CMI and sarcopenia and CMM, cox proportional risk regression models were used.

**Results:**

The median age of all participants was 57 years, with 47.1% being male. Over the 8-year follow-up, 1,362 individuals developed CMM. The incidence of CMM was 8.7/1,000 person-years in the group without sarcopenia or high CMI, 17.37/1,000 person-years in those with high CMI, 14.22/1,000 person-years in the sarcopenia group, and 22.34/1,000 person-years in the group with both conditions. After adjusting for covariates, the group with both sarcopenia and high CMI had a significantly increased risk of CMM (HR 2.48, 95% CI 1.12-5.51) and heart disease (HR 2.04, 95% CI 1.05-3.98). Among those over 65 years, sarcopenia was discovered to be associated with an increased risk of CMM [HR (95% CI: 4.83 (1.22, 19.06)]. The risk of CMM was further increased to 7.31-fold (95% CI:1.72, 31.15) when combined with high CMI.

**Conclusions:**

The combination of sarcopenia and high CMI is associated with an increased risk of developing CMM. Early identification and intervention of sarcopenia and CMI not only enable the development of targeted therapeutic strategies but also provide potential opportunities to reduce the morbidity and mortality of CMM.

## Introduction

With the increasing global urbanization and changes in lifestyles and diet, the prevalence of cardiometabolic diseases is on the rise, presenting a significant public health challenge worldwide (1). Several large-scale population-based cohort studies have identified a high degree of comorbidity between cardiovascular disease and abnormal glucose metabolism, which increases the risk of cardiovascular disease (2–4). The EUROASPIRE IV study conducted in Europe found that approximately 25% of patients with coronary heart disease patients who had not been previously diagnosed with diabetes were newly diagnosed with the condition. Additionally, 46% to 66% were found to be in the pre-diabetes stage, while only 10.5% to 26.6% had completely normal glucose metabolism (5). In China, it is estimated that approximately 500,000 cardiovascular deaths per year can be attributed to diabetes, making the co-morbidity of cardiovascular disease and diabetes/pre-diabetes a significant concern. Scientists have introduced the concept of ‘cardiometabolic multimorbidity (CMM)’ to describe this phenomenon (6). CMM is defined as the simultaneous occurrence of two or more cardiovascular metabolic diseases (CMD) (7). In the United States, the prevalence of CMM is 14.4%, and it is higher in men and older adults (8). It is worth noting that the health implications associated with CMM are considerably more severe than those associated with single CMD (9). Older adults with any cardiometabolic disease or two cardiometabolic diseases had a significantly shorter life expectancy of 7 and 12 years, respectively, compared to those without heart disease (10). Furthermore, individuals with one CMD or CMM were 1.41 and 1.89 times more likely to experience higher levels of mental stress than those without cardiometabolic disease (11). Therefore, the early prevention and treatment of CMM remain a critical challenge that requires further attention.

Sarcopenia is a syndrome that is characterized by a progressive age-related decline in skeletal muscle mass (SMM) and strength, which leads to dysfunction in the body. According to the 2019 report from the European Working Group on Sarcopenia in Older People (EWGSOP), the prevalence of sarcopenia is 6%-12% globally, 14%-33% in older adults aged 65 years and older, and as high as 78% among hospitalized patients (12). The 2019 report by the Asian Sarcopenia Working Group indicates that the prevalence of sarcopenia in the Asian older adult population ranges from 5.5% to 25.7% (13). There is increasing evidence linking sarcopenia to various adverse outcomes, such as falls, debility, frequent medical visits, and a higher risk of mortality (14). Sarcopenia has also been identified as a risk factor for the development of cardiovascular disease (15). A comprehensive study conducted in a Chinese population found a significant association between sarcopenia and an increased risk of cardiovascular disease, including both heart disease and stroke (16). Sarcopenia often coexists with an accumulation of adipose tissue, particularly abdominal obesity (17). Severe muscle loss and obesity can synergistically increase metabolic disturbances and the risk of adverse outcomes such as myocardial infarction, stroke, and death more than sarcopenia or simple obesity alone (18). Obesity is recognized as a key modifiable risk factor for CMM (19). Although high Body Mass Index (BMI) is currently a widely used international metric for assessing the extent of human obesity, it does not accurately reflect body composition or the distribution of visceral fat. Furthermore, it should be noted that BMI is not a reliable predictor of cardiovascular disease risk in certain populations. Body fat distribution, rather than the degree of overall obesity, leads to greater cardiovascular risk (20). In 2015, Ichiro et al. proposed the Cardiometabolic Index (CMI) as an obesity-related anthropometric index which combines height (m) and waist circumference (m) and offers a more precise representation of a person’s body shape than traditional indicators such as Body Mass Index (BMI), waist circumference, and hip circumference (21). Several studies have demonstrated a significant association between and hypertension, carotid atherosclerosis, and diabetes mellitus (22–24). However, previous studies have primarily focused on the association of sarcopenia or CMI with a single CMD and have mostly employed cross-sectional survey methods instead of prospective studies with a higher level of evidence. Unfortunately, there are no comprehensive studies that investigate the relationship between sarcopenia and/or CMI and CMM. Consequently, substantial knowledge gaps and uncertainties persist regarding the interactions and potential mechanisms between sarcopenia and/or CMI and CMM.

This study aims to investigate the association between sarcopenia and CMI, both alone and in combination with CMM. The goal is to determine whether the coexistence of sarcopenia and CMI increases the risk of developing CMM. The findings will provide a solid and reliable foundation for clinical prevention and treatment strategies, and may facilitate the medical community make new breakthroughs in related fields.

## Methods

### Study design and population

The China Longitudinal Study of Health and Retirement (CHARLS)is a long-term survey that documents the social, economic, and health status of middle-aged and older adults in China aged 45 and above, along with their families. The study enrolled 17,708 participants in 2011, with follow-up surveys conducted every two years. Ethical approval for CHARLS was obtained from the Ethical Review Committee of Peking University(IRB 00001052 -11015), and all participants provided informed consent. When performing research involving human subjects, all procedures were following institutional and national research council ethical standards as well as the Declaration of Helsinki and its subsequent amendments or similar ethical standards. For further details and raw data, please visit https://charls.pku.edu.cn.

**Figure 1:**
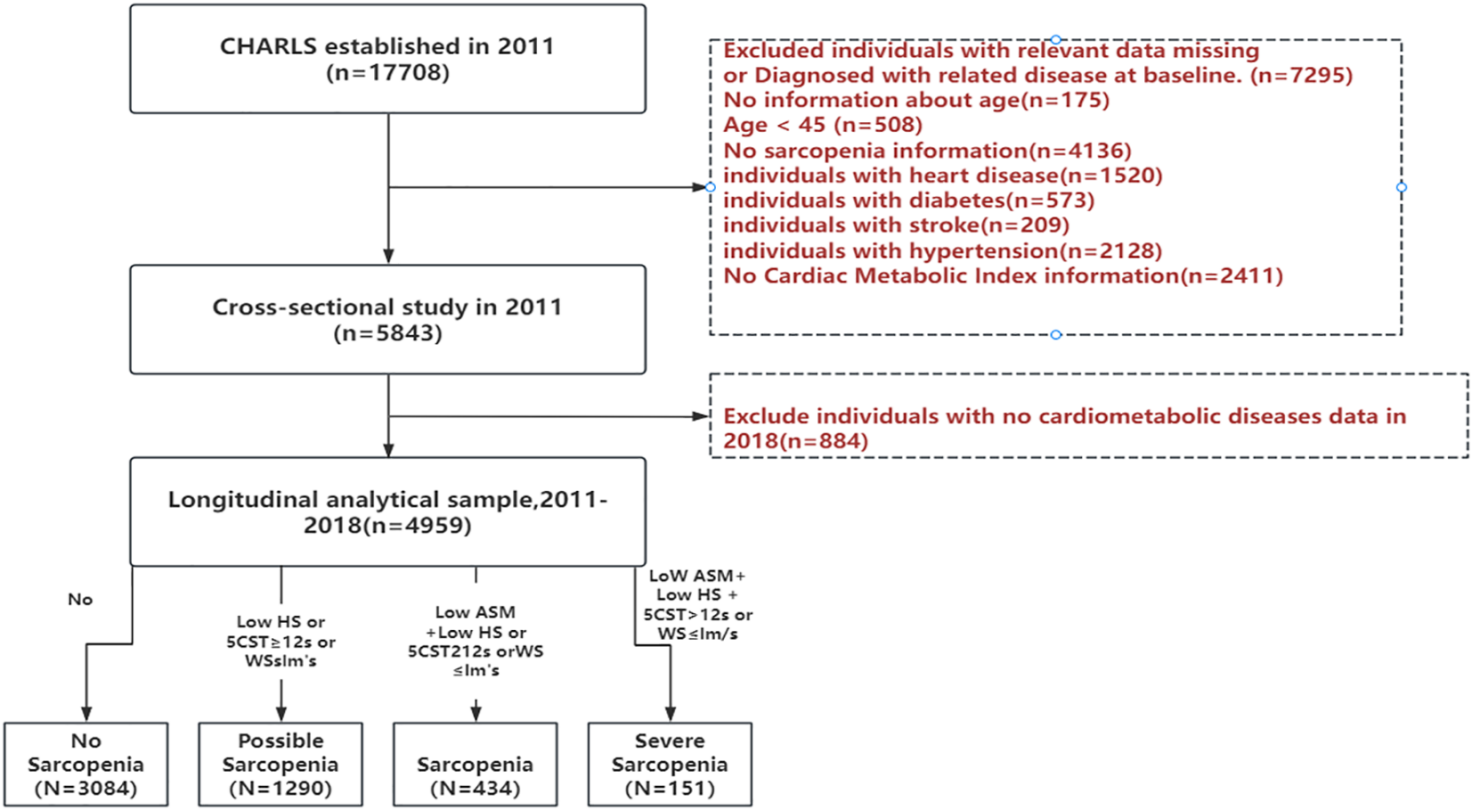
Flowchart for participants of this current study.

This study commenced in 2011 with a base sample of 17,708 respondents and four follow-up surveys. To guarantee the dependability and comprehensiveness of our results, we established the following exclusion criteria. These criteria excluded 1) individuals with incomplete data or a diagnosis of a specific illness (heart disease, diabetes, stroke, hypertension); 2) those with unknown age information; 3) those without gender-related details; and 4) those lacking sarcopenia-related data. After a rigorous screening process, our final analytical sample consisted of 4959 participants. None of the individuals had cardiovascular-metabolic diseases at the time of the 2011 CHARLS survey and continued to participate in the 2013, 2015, and 2018 follow-up surveys. The detailed selection process is outlined in **Figure 1**, providing a visual representation of the screening procedures.

### Definition of sarcopenia

The Asian Working Group on Sarcopenia (AWGS) 2019 criteria were used to assess sarcopenia. The measurements included grip strength, gait speed, the five-chair rise test, the Simple Physical Performance Battery (SPPB) and appendicular skeletal muscle mass (ASM) values. Grip strength served as the primary indicator of muscle strength, with cutoff values of <28 kg for men and <18 kg for women. To estimate muscle mass, we used the ASM formula, which considers weight, height, sex, and age. Previous studies have demonstrated high agreement between the ASM formula and double x-ray absorptiometry (DXA). The threshold for low muscle mass was based on the lowest 20% of gender-specific height-adjusted muscle mass (ASM/Ht^2^) in the study population (25). Therefore, we considered ASM/Ht^2^ values <5.27 kg/m^2^ for females and ASM/Ht2 <7.01 kg/m^2^ for males as indicators of low muscle mass. The assessment of somatic functioning included gait speed, the five-chair rise test, and SPPB scores. Reduced somatic function were defined as gait speed less than 1.0 m/s, the five-chair rise test taking 12 seconds or more, or an SPPB score less than 9. The study cohort comprised 3084 non-sarcopenia, 1290 possible sarcopenia, 434 sarcopenia, and 151 severe sarcopenia cases. For the purpose of this study, individuals diagnosed with sarcopenia and severe sarcopenia were grouped together. This decision was made to ensure adequate sample size for statistical analysis and to reflect the shared clinical significance and physiological characteristics of these conditions in the context of increased risk for cardiometabolic multimorbidity.

### Cardiac metabolic index

CMI = TG/HDL-C × WHtR, TG: triglycerides, HDL-C: high-density lipoprotein cholesterol, WHtR: waist circumference to height ratio. Both waist circumference and height are measured in centimeters. To classify CMI, we used an interquartile approach due to the lack of a uniform classification standard. We selected the 75th percentile as the cutoff point based on the distribution of CMI in the study population. Individuals with CMI above the 75th quartile were defined as having ‘high CMI’. This aim of this classification was to identify individuals with high CMI levels for further investigation of their association with CMM. This will enable a more accurate assessment of an individual’s cardio-metabolic risk and provide a basis for developing appropriate interventions.

### Definitions of CMM

CMM is defined as the coexistence of two or more cardiovascular and metabolic diseases, such as hypertension, diabetes, heart disease, and stroke. To determine whether an individual has any of these diseases, we ask, ‘Has your doctor informed you that you have hypertension?’, ‘Have you been diagnosed with diabetes mellitus or elevated blood glucose levels (including abnormal glucose tolerance and raised fasting blood glucose)?’, ‘Have you been with heart disease (such as myocardial infarction, coronary heart disease, angina pectoris, congestive heart failure, or other heart conditions)?’, and ‘Have you experienced a stroke (including cerebral infarction and cerebral hemorrhage) as diagnosed by a doctor?’.

### Covariates

This study collected baseline data on socio-demographic status and health-related information using a structured questionnaire. The data aimed to provide a comprehensive overview of the socio-demographic characteristics and health status. Gender, age, education level, marital status, place of residence, and exercise habits were collected as socio-demographic variables. These variables are important for understanding the social background and lifestyle of the subjects, which can help identify potential correlations with their health status. Health-related factors include physiological indicators such as BMI, smoking and drinking status, blood pressure levels, blood counts, CRP, lipids, renal function, fasting blood glucose, and uric acid.

### Statistical analysis

In the follow-up study, participants were subdivided into four groups based on their health status: 1) no sarcopenia and normal CMI, 2) high CMI only, 3) sarcopenia only, and 4) both sarcopenia and high CMI. To explore the differences in baseline characteristics among the groups, we flexibly used chi-square (χ2) tests as well as the Pearson χ2 test or Fisher exact test. To assess the potential association between sarcopenia and CMI status at baseline and subsequent CMM events, we applied the Cox proportional risk model to calculate the corresponding hazard ratios (HR) and 95% confidence intervals.

For the longitudinal data analysis, we constructed three increasingly comprehensive models. Model 1 served as the unadjusted baseline model. Model 2 was adjusted based on Model 1 by incorporating age and gender as covariates. Model 3 further integrated additional adjustments based on Model 2, including education, residence, marital status, smoking habits, alcohol consumption, body mass index (BMI), systolic blood pressure (SBP), diastolic blood pressure (DBP), white blood cell count, hemoglobin, platelet count, C-reactive protein, total cholesterol, low-density lipoprotein (LDL), blood urea nitrogen (BUN), creatinine, blood glucose, and uric acid. Statistical analysis was conducted using R studio (version 4.3.2). A P value less than 0.05 was considered statistically significant.

## Results

### Characteristics of the study participants

**Table 1** presents the characteristics of the participants based on the quartiles of CMI (Q1 <0.27 mmol/l, 0.27 <Q2 <0.44 mmol/l, 0.44 <Q3 <0.75 mmol/l, Q4 >0.75 mmol/l). After screening by exclusion criteria, a total of 4959 participants aged 45 or above and free of cardiometabolic disease events at baseline were included. The median age of the study population was 57(50.8 63), and 2338 (47.1%) were male. Participants with higher CMI levels were more likely to be female, reside in rural areas, have lower levels of education, have a higher BMI, and engage in less physical activity. Additionally, they also had higher levels of C-reactive protein (CRP), blood glucose, total cholesterol (TC), triglycerides (TG), as well as higher levels of low-density lipoprotein cholesterol (LDL-C). According to AWGS 2019 criteria, the cohort comprised 3084(62.1%) non-sarcopenia, 1290(26%) possible sarcopenia, 434(8.8%) sarcopenia and 151(3%) severe sarcopenia cases. Over the 8-year follow-up period, there were 1362 individuals who developed CMM and 3108 individuals who developed one type of CMD.

**Table 1:** Characteristics of 4959 participants by CMI.

Characteristics	Q1<0.27 mmol/l	0.27<Q2<0.44 mmol/l	0.44<Q3<0.75 mmol/l	Q4>0.75 mmol/l	Total	P value
Total	1239	1240	1240	1240	4959	
Gender						< 0.001
Female	573 (46.2)	653 (52.7)	708 (57.1)	687 (55.4)	2621 (52.9)	
Male	666 (53.8)	587 (47.3)	532 (42.9)	553 (44.6)	2338 (47.1)	
Age						0.005
median (IQR)	57 (51,64)	57 (50,63)	57 (51,64)	56 (50,62)	57 (50.8,63)	
BMI						< 0.001
median (IQR)	21.1 (19.4,22.9)	22.1 (20.3,24)	23.2 (21.2,25.5)	24.6 (22.5,27.1)	22.7 (20.6,25)	
Education						0.048
low	861 (69.5)	854 (68.9)	876 (70.6)	817 (65.9)	3408 (68.7)	
middle	372 (30)	373 (30.1)	352 (28.4)	404 (32.6)	1501 (30.3)	
high	6 (0.5)	13 (1)	12 (1)	19 (1.5)	50 (1)	
Marriage						0.591
No	21 (1.8)	15 (1.3)	14 (1.2)	19 (1.6)	69 (1.5)	
Yes	1163 (98.2)	1168 (98.7)	1158 (98.8)	1147 (98.4)	4636 (98.5)	
Rural						< 0.001
Urban	329 (26.6)	353 (28.5)	431 (34.8)	476 (38.4)	1589 (32)	
Rural	910 (73.4)	887 (71.5)	809 (65.2)	764 (61.6)	3370 (68)	
strenuous exercise						< 0.001
No	259 (47.9)	279 (54)	310 (61)	344 (63.8)	1192 (56.6)	
Yes	282 (52.1)	238 (46)	198 (39)	195 (36.2)	913 (43.4)	
moderate intensity exercise						0.027
No	176 (32.5)	200 (38.7)	201 (39.6)	219 (40.7)	796 (37.8)	
Yes	365 (67.5)	317 (61.3)	307 (60.4)	319 (59.3)	1308 (62.2)	
light intensity exercise						0.59
No	92 (17.1)	95 (18.4)	94 (18.5)	109 (20.3)	390 (18.6)	
Yes	447 (82.9)	421 (81.6)	413 (81.5)	427 (79.7)	1708 (81.4)	
Smoking						0.001
No	697 (56.3)	753 (60.7)	792 (63.9)	764 (61.6)	3006 (60.6)	
Yes	542 (43.7)	487 (39.3)	448 (36.1)	476 (38.4)	1953 (39.4)	
Drinking						< 0.001
No	668 (54)	768 (62)	793 (64)	797 (64.3)	3026 (61.1)	
Yes	570 (46)	471 (38)	446 (36)	443 (35.7)	1930 (38.9)	
SBP, mmHg						< 0.001
median (IQR)	119 (108,130.5)	119.5 (109.8,131)	123 (112.5,135.2)	124.5 (113.8,137.5)	121.5 (110.5,134)	
DPB, mmHg						< 0.001
mean (SD)	70.5 (63.5,77.5)	71.5 (64.5,79)	73 (66.5,81)	74.5 (67.5,82)	72.5 (65.5,80)	
White Blood Cell, 10^9^/L						< 0.001
median (IQR)	5.6 (4.7,6.7)	5.9 (4.8,7.1)	6 (5,7.1)	6.2 (5.2,7.5)	5.9 (4.9,7.1)	
Hemoglobin, g/dL						< 0.001
median (IQR)	14 (12.8,15.2)	14.1 (12.9,15.3)	14.2 (13,15.4)	14.5 (13.2,15.8)	14.2 (13,15.5)	
Platelets, 10^9^/L						0.005
median (IQR)	202 (162,248)	210 (163.2,259)	206 (162,254)	213 (163,264)	208 (163,255)	
CRP , mg/L						< 0.001
median (IQR)	0.7 (0.4,1.4)	0.8 (0.5,1.7)	0.9 (0.5,1.8)	1.2 (0.7,2.3)	0.9 (0.5,1.8)	
HDL-c, mg/dL						< 0.001
median (IQR)	65.7 (57.2,75.2)	54.9 (48.3,61.9)	47.9 (42.1,53.7)	37.5 (32.5,43.7)	50.6 (41.7,61.1)	
Total Cholesterol, mg/dL						< 0.001
median (IQR)	184 (163.9,207.6)	187.1 (164.2,209.5)	191 (166.6,213.4)	194.8 (170.9,222.7)	189.4 (166.2,213.4)	
LDL-c, mg/dL						< 0.001
median (IQR)	107.5 (89.3,127.1)	116.4 (95.9,138.8)	119.5 (99.2,140.7)	112.7 (87.8,135.3)	113.7 (93.2,135.3)	
Triglycerides						< 0.001
median (IQR)	60.2 (49.6,70.8)	85 (74.3,98.2)	114.2 (100,133.6)	193.4 (154,257.5)	100 (71.7,144.3)	
BUN, mg/dl						< 0.001
median (IQR)	16 (13.3,19)	15.1 (12.5,18.5)	14.8 (12.4,17.7)	14.7 (12.2,17.4)	15.2 (12.5,18.2)	
Creatinine, mg/dl						0.011
median (IQR)	0.7 (0.6,0.9)	0.7 (0.6,0.9)	0.7 (0.6,0.9)	0.8 (0.7,0.9)	0.7 (0.6,0.9)	
Glucose, mg/dl						< 0.001
median (IQR)	98.6 (91.6,106.7)	99.5 (93.1,107.8)	100.6 (93.6,109.5)	105.8 (97.4,117.6)	101.2 (93.8,110.2)	
Uric Acid, mg/dl						< 0.001
median (IQR)	4.1 (3.4,4.9)	4 (3.4,4.8)	4.2 (3.5,5)	4.5 (3.8,5.4)	4.2 (3.5,5)	
Sarcopenia						< 0.001
No	773 (62.4)	757 (61)	747 (60.2)	807 (65.1)	3084 (62.2)	
Possible	242 (19.5)	312 (25.2)	367 (29.6)	369 (29.8)	1290 (26)	
Severe	51 (4.1)	41 (3.3)	39 (3.1)	20 (1.6)	151 (3)	
Yes	173 (14)	130 (10.5)	87 (7)	44 (3.5)	434 (8.8)	
Number of CMM						< 0.001
0	69 (5.6)	105 (8.5)	142 (11.5)	173 (14)	489 (9.9)	
1	865 (69.8)	819 (66)	744 (60)	680 (54.8)	3108 (62.7)	
>=2	305 (24.6)	316 (25.5)	354 (28.5)	387 (31.2)	1362 (27.5)	

### The longitudinal association of sarcopenia and CMI and CMM

**Table 2** shows the relationship between CMI, sarcopenia, and CMM incident. After adjusting for socio-demographic characteristics and health-related factors, higher CMI values were associated with an increased risk of heart disease [HR (95%CI): 1.13(0.78, 1.49)], stroke [HR (95%CI): 1.65(1.07, 2.60)], hypertension [HR (95%CI): 1.11(0.92, 1.33)], CMM [HR (95%CI): 1.48(0.92, 1.33)], and diabetes [HR (95%CI): 1.89(1.33, 2.68)]. Notably, CMI had the strongest association with an elevated risk of diabetes and CMM development, at 1.89-fold and 1.48-fold respectively. However, after adjusting for covariates, there was no statistically difference in risk between those with and without sarcopenia for developing CMM, diabetes, heart disease, stroke, and hypertension.

**Table 2:** Associations of the CMI and sarcopenia with the risk of CMM.

Factors	HR(95%CI)				
	Diabetes	Heart disease	Stroke	Hypertension	CMM
**Sarcopenia**	**0.96 (0.46,1.96)**	**1.52 (0.87,1.48)**	**0.84 (0.35,2.03)**	**1.31 (0.90,1.89)**	**1.76 (0.95,3.26)**
**CMI**	1.89 (1.33,2.68)***	**1.13 (0.78,1.49)**	1.67 (1.07,2.60)*	**1.11 (0.92,1.33)**	1.48 (1.09,2.01)*
**P for interaction**	**0.61**	**0.06**	**0.97**	**0.45**	**0.39**

Low CMI and no-sarcopenia were used as control groups.

HR, hazard ratio; CMM, Cardiometabolic multimorbidity; CMI, cardiac metabolic index;

Model: adjusted age, sex, residence, education, marital status, smoking, drinking, BMI, SBP, DBP, strenuous exercise, moderate intensity exercise, White Blood cell, Hemoglobin, Platelets, CRP, Total Cholesterol, LDL-c, BUN, Creatinine, Glucose, Uric Acid.

* indicates P<0.05, *** indicates P<0.001.

**Figure 2:**
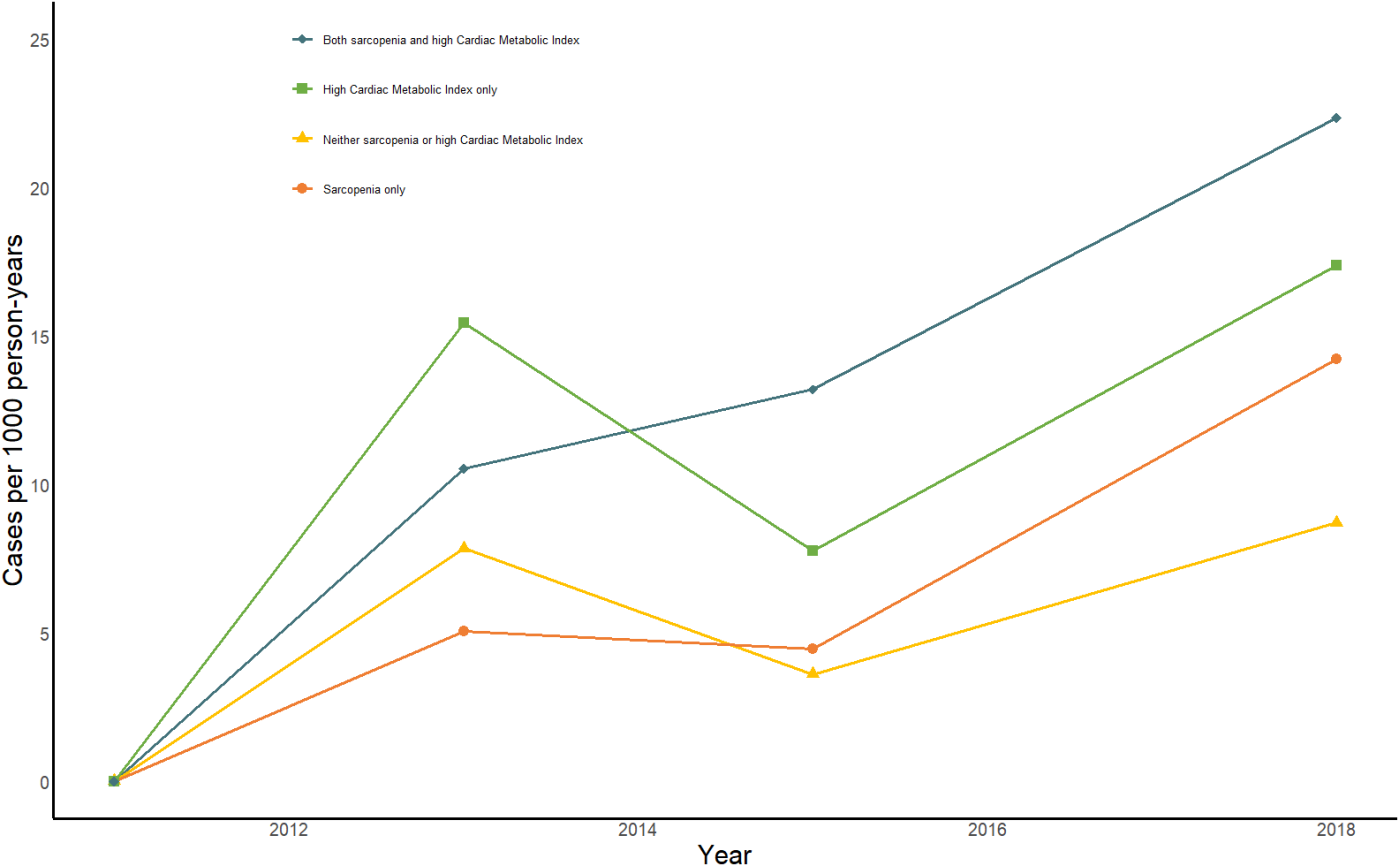
Impact of sarcopenia, high CMI, and sarcopenia combined with high CMI on person-year incidence of CMM from 2011-2018. Bule line: Sarcopenia combined with high CMI; Green line: high CMI only; Yellow line: No sarcopenia or high CMI; orange line: Sarcopenia only.

**Figure 2** displays the change in annual incidence of CMM among all participants from 2011 to 2018. Our findings suggest that the incidence of CMM may significantly in individuals with sarcopenia and high CMI, either alone or in combination. The group with sarcopenia combined with high CMI had the highest prevalence at all time points and showed a significant upward trend. This result highlights the significant threat to individual health posed by the combination of sarcopenia and high CMI. **Table 3** shows the relationships between different combinations of sarcopenia, CMI, and CMM components. In the longitudinal analyses, the prevalence of CMM was 8.7/1000 person-years in the group with neither sarcopenia nor high CMI, 17.37/1000 person-years in the high CMI group, 14.22/1000 person-years in the sarcopenia group, and 22.34/1000 person-years in the group with both sarcopenia reduction and high CMI. Model 1 found that the group with sarcopenia and high CMI group had an increased risk of CMM, heart disease, diabetes, stroke, and hypertension. After adjusting for age and sex in Model 2, the group with sarcopenia and high CMI still had an increased risk of CMM, diabetes, and stroke. After adjusting for all covariates in model 3, the combination of sarcopenia and high CMI was found to be associated with an increased risk of heart disease [HR (95% CI): 2.04 (1.05, 3.98)] and CMM [HR (95% CI): 2.48 (1.12, 5.51)], compared to those with neither sarcopenia nor CMI. However, no significant associations were found with the attenuated outcomes of diabetes, stroke, and hypertension. High CMI was only associated with a 1.86-fold (95% CI: 1.14,3.01) probability of developing diabetes. There were no significant longitudinal associations between sarcopenia alone and different components of CMM.

**Table 3:** Associations of the CMI and sarcopenia with the incidence of CMM.

Factors	cases	Incidence rate(per 1000 person-year)	Model 1 HR (95%CI)	Model 2 HR (95%CI)	Model 3 HR (95%CI)
Heart disease					
**Neither sarcopenia or high Cardiac Metabolic Index**	**159**	**15.19**	**Ref.**	**Ref.**	**Ref.**
**High Cardiac Metabolic Index only**	**193**	**18.27**	**1.21 (0.98,1.49)**	**1.19 (0.97,1.47)**	**1.15 (0.78,1.70)**
**Sarcopenia only**	**60**	**22.42**	1.50 (1.11,2.02)**	**1.15 (0.83,1.59)**	**1.41 (0.80,2.51)**
**Both sarcopenia and high Cardiac Metabolic Index**	**38**	**30.11**	2.04 (1.44,2.91)***	**1.40 (0.94,2.09)**	2.04 (1.05,3.98)*
Diabetes					
**Neither sarcopenia or high Cardiac Metabolic Index**	**69**	**6.5**	**Ref.**	**Ref.**	**Ref.**
**High Cardiac Metabolic Index only**	**188**	**17.74**	2.80 (2.12,3.68)***	2.78 (2.11,3.66)***	1.86 (1.14,3.01)*
**Sarcopenia only**	**26**	**9.48**	**1.47 (0.94,2.31)**	**1.52 (0.93,2.46)**	**0.84 (0.32,2.23)**
**Both sarcopenia and high Cardiac Metabolic Index**	**18**	**13.92**	2.17 (1.29,3.65)**	2.22 (1.26,3.92)**	**2.18 (0.88,5.41)**
Stroke					
**Neither sarcopenia or high Cardiac Metabolic Index**	**46**	**4.3**	**Ref.**	**Ref.**	**Ref.**
**High Cardiac Metabolic Index only**	**76**	**7.02**	1.64 (1.14,2.37)**	1.70 (1.18,2.45)**	**1.31 (0.69,2.48)**
**Sarcopenia only**	**23**	**8.35**	1.97 (1.19,3.24)**	**1.41 (0.81,2.43)**	**0.77 (0.27,2.19)**
**Both sarcopenia and high Cardiac Metabolic Index**	**18**	**13.71**	3.28 (1.90,5.65)***	2.27 (1.22,4.21)**	**1.32 (0.41,4.27)**
Hypertension					
**Neither sarcopenia or high Cardiac Metabolic Index**	**323**	**31.43**	**Ref.**	**Ref.**	**Ref.**
**High Cardiac Metabolic Index only**	**454**	**44.79**	1.47 (1.27,1.69)***	1.49 (1.29,1.72)***	**0.98 (0.76,1.26)**
**Sarcopenia only**	**93**	**37.76**	**1.22 (0.97,1.53)**	**0.94 (0.74,1.21)**	**1.23 (0.80,1.89)**
**Both sarcopenia and high Cardiac Metabolic Index**	**59**	**47.23**	1.56 (1.18,2.06)**	**1.15 (0.85,1.56)**	**1.43 (0.87,2.36)**
CMM					
**Neither sarcopenia or high Cardiac Metabolic Index**	**93**	**8.7**	**Ref.**	**Ref.**	**Ref.**
**High Cardiac Metabolic Index only**	**186**	**17.37**	2.04 (1.59,2.61)***	2.06 (1.61,2.65)***	**1.28 (0.81,2.03)**
**Sarcopenia only**	**39**	**14.22**	1.65 (1.14,2.40)**	**1.31 (0.87,1.96)**	**1.71 (0.82,3.55)**
**Both sarcopenia and high Cardiac Metabolic Index**	**29**	**22.34**	2.66 (1.75,4.03)***	2.01 (1.26,3.20)**	2.48 (1.12,5.51)*

HR, hazard ratio; CMM, Cardiometabolic multimorbidity; CMI, cardiac metabolic index;

Model 1: unadjusted;

Model 2: adjusted for age, sex;

Model 3: adjusted as model 2 with further adjustment for residence, education, marital status, smoking, drinking, BMI, SBP, DBP, strenuous exercise, moderate intensity exercise, White Blood cell, Hemoglobin, Platelets, CRP, Total Cholesterol, LDL-c, BUN, Creatinine, Glucose, Uric Acid. * indicates P<0.05, ** indicates P<0.01, *** indicates P<0.001.

### Subgroup analysis

Among males, high CMI was found to be associated with an increased risk of stroke [HR (95% CI): 3.15 (1.24, 8.01)]. The risk of stroke was further increased to 5.85-fold [95% CI: 1.20, 25.59] when comorbid sarcopenia was present. In females, the combination of sarcopenia and high CMI was associated with a higher risk of heart disease [HR (95% CI): 2.75 (1.18, 6.41)]. The longitudinal association between sarcopenia combined with high CMI and CMM was not significant for both genders (**Table 4**). However, subgroup analyses stratified by age reflected distinct patterns of association. Among those aged below 65 years, sarcopenia combined with high CMI was associated with an elevated risk of heart disease only [HR (95% CI): 2.95 (1.18, 7.40)], while the longitudinal association with CMM was not significant after adjusting for model 2. In contrast, for those aged 65 years or older, sarcopenia was found to be associated with a higher risk of CMM [HR (95% CI): 4.83 (1.22, 19.06)]. This risk was further elevated to 7.31-fold (95% CI: 1.72, 31.15) when combined with high CMI (**Table 5**). These findings suggest that the association between sarcopenia and high CMI with CMM may vary depending on age, and that the association appears to be more pronounced in the older adult population.

**Table 4:** Sex Subgroup Analysis of the Effect of CMI and sarcopenia on CMM.

Factors		Model 1 HR(95%CI)	Model 2 HR(95%CI)
**Male**	Heart disease		
	**Neither sarcopenia or high Cardiac Metabolic Index**	**Ref.**	**Ref.**
	**High Cardiac Metabolic Index only**	1.64(1.18,2.27)**	**1.57(0.87,2.85)**
	**Sarcopenia only**	1.87(1.15,3.03)*	**1.54(0.66,3.60)**
	**Both sarcopenia and high Cardiac Metabolic Index**	**1.52(0.69,3.33)**	**0.75(0.16,3.53)**
	Diabetes		
	**Neither sarcopenia or high Cardiac Metabolic Index**	**Ref.**	**Ref.**
	**High Cardiac Metabolic Index only**	3.32(2.16,5.11)***	**1.54(0.67,3.54)**
	**Sarcopenia only**	**1.68(0.81,3.46)**	**1.37(0.26,7.28)**
	**Both sarcopenia and high Cardiac Metabolic Index**	3.84(1.75,8.46)***	**3.74(0.73,19.19)**
	Stroke		
	**Neither sarcopenia or high Cardiac Metabolic Index**	**Ref.**	**Ref.**
	**High Cardiac Metabolic Index only**	2.37(1.43,3.94)***	3.15(1.24,8.01)*
	**Sarcopenia only**	**1.72(0.76,3.89)**	**2.31(0.61,8.77)**
	**Both sarcopenia and high Cardiac Metabolic Index**	5.00(2.21,11.28)***	5.85(1.20,28.59)*
	Hypertension		
	**Neither sarcopenia or high Cardiac Metabolic Index**	**Ref.**	**Ref.**
	**High Cardiac Metabolic Index only**	1.48(1.22,1.81)***	**0.91(0.64,1.31)**
	**Sarcopenia only**	**1.25(0.90,1.74)**	**1.16(0.62,2.16)**
	**Both sarcopenia and high Cardiac Metabolic Index**	**1.33(0.81,2.19)**	**1.75(0.75,4.09)**
	CMM		
	**Neither sarcopenia or high Cardiac Metabolic Index**	**Ref.**	**Ref.**
	**High Cardiac Metabolic Index only**	2.75(1.90,3.96)***	**1.50(0.79,2.86)**
	**Sarcopenia only**	**1.74(0.96,3.16)**	**1.89(0.71,5.05)**
	**Both sarcopenia and high Cardiac Metabolic Index**	3.40(1.70,6.81)***	**2.17(0.61,7.71)**
**Female**	Heart disease		
	**Neither sarcopenia or high Cardiac Metabolic Index**	**Ref.**	**Ref.**
	**High Cardiac Metabolic Index only**	**0.98(0.74,1.29)**	**0.79(0.46,1.36)**
	**Sarcopenia only**	**1.36(0.94,1.95)**	**1.48(0.70,3.17)**
	**Both sarcopenia and high Cardiac Metabolic Index**	1.82(1.19,2.78)**	2.75(1.18,6.41)*
	Diabetes		
	**Neither sarcopenia or high Cardiac Metabolic Index**	**Ref.**	**Ref.**
	**High Cardiac Metabolic Index only**	2.22 (1.56,3.17)***	**1.78 (0.96,3.28)**
	**Sarcopenia only**	**1.33 (0.77,2.29)**	**1.22 (0.38,3.89)**
	**Both sarcopenia and high Cardiac Metabolic Index**	**1.16 (0.54,2.45)**	**1.77 (0.53,5.90)**
	Stroke		
	**Neither sarcopenia or high Cardiac Metabolic Index**	**Ref.**	**Ref.**
	**High Cardiac Metabolic Index only**	**1.19 (0.68,2.07)**	**0.55 (0.19,1.59)**
	**Sarcopenia only**	2.19 (1.15,4.21)*	**0.11 (0.01,1.10)**
	**Both sarcopenia and high Cardiac Metabolic Index**	2.87 (1.37,6.03)**	**0.31 (0.04,2.13)**
	Hypertension		
	**Neither sarcopenia or high Cardiac Metabolic Index**	**Ref.**	**Ref.**
	**High Cardiac Metabolic Index only**	1.43 (1.16,1.75)***	**1.01 (0.69,1.48)**
	**Sarcopenia only**	**1.19 (0.88,1.62)**	**1.28 (0.69,2.38)**
	**Both sarcopenia and high Cardiac Metabolic Index**	1.73 (1.22,2.44)**	**1.36 (0.70,2.63)**
	CMM		
	**Neither sarcopenia or high Cardiac Metabolic Index**	**Ref.**	**Ref.**
	**High Cardiac Metabolic Index only**	1.67 (1.17,2.36)**	**1.02 (0.52,2.02)**
	**Sarcopenia only**	1.72 (1.08,2.77)*	**2.18 (0.72,6.67)**
	**Both sarcopenia and high Cardiac Metabolic Index**	2.23 (1.29,3.86)**	**2.24 (0.72,7.04)**

HR, hazard ratio; CMM, Cardiometabolic multimorbidity; CMI, cardiac metabolic index;

Model 1: unadjusted;

Model 2: adjusted age, sex, residence, education, marital status, smoking, drinking, BMI, SBP, DBP, strenuous exercise, moderate intensity exercise, White Blood cell, Hemoglobin, Platelets, CRP, Total Cholesterol, LDL-c, BUN, Creatinine, Glucose, Uric Acid. * indicates P<0.05, ** indicates P<0.01, *** indicates P<0.001.

**Table 5:** Age Subgroup Analysis of the Effect of CMI and sarcopenia on CMM.

Factors		Model 1 HR(95%CI)	Model 2 HR(95%CI)
**Age<65**	Heart disease		
	**Neither sarcopenia or high Cardiac Metabolic Index**	**Ref.**	**Ref.**
	**High Cardiac Metabolic Index only**	**1.18 (0.94,1.47)**	**1.17 (0.77,1.77)**
	**Sarcopenia only**	**0.88 (0.51,1.52)**	**0.92 (0.38,2.23)**
	**Both sarcopenia and high Cardiac Metabolic Index**	2.79 (1.58,4.92)***	2.95 (1.18,7.40)*
	Diabetes		
	**Neither sarcopenia or high Cardiac Metabolic Index**	**Ref.**	**Ref.**
	**High Cardiac Metabolic Index only**	2.63 (1.98,3.49)***	**1.53 (0.94,2.50)**
	**Sarcopenia only**	**1.73 (0.95,3.13)**	**1.12 (0.33,3.85)**
	**Both sarcopenia and high Cardiac Metabolic Index**	**1.21 (0.38,3.83)**	**2.18 (0.60,7.93)**
	Stroke		
	**Neither sarcopenia or high Cardiac Metabolic Index**	**Ref.**	**Ref.**
	**High Cardiac Metabolic Index only**	1.60 (1.07,2.37)*	**1.42 (0.68,2.95)**
	**Sarcopenia only**	**1.55 (0.70,3.47)**	**1.04 (0.22,4.90)**
	**Both sarcopenia and high Cardiac Metabolic Index**	**1.36 (0.33,5.62)**	**1.49 (0.18,12.64)**
	Hypertension		
	**Neither sarcopenia or high Cardiac Metabolic Index**	**Ref.**	**Ref.**
	**High Cardiac Metabolic Index only**	1.50 (1.28,1.75)***	**1.03 (0.78,1.36)**
	**Sarcopenia only**	**0.89 (0.60,1.31)**	**1.09 (0.54,2.22)**
	**Both sarcopenia and high Cardiac Metabolic Index**	**0.76 (0.38,1.54)**	**0.78 (0.24,2.54)**
	CMM		
	**Neither sarcopenia or high Cardiac Metabolic Index**	**Ref.**	**Ref.**
	**High Cardiac Metabolic Index only**	1.91 (1.47,2.48)***	**1.34 (0.82,2.20)**
	**Sarcopenia only**	**1.22 (0.67,2.23)**	**0.74 (0.17,3.22)**
	**Both sarcopenia and high Cardiac Metabolic Index**	**0.61 (0.15,2.49)**	**1.72 (0.38,7.90)**
**Age≥65**	Heart disease		
	**Neither sarcopenia or high Cardiac Metabolic Index**	**Ref.**	**Ref.**
	**High Cardiac Metabolic Index only**	**1.47 (0.82,2.62)**	**1.05 (0.26,4.19)**
	**Sarcopenia only**	**1.65 (0.97,2.80)**	**2.00 (0.69,5.74)**
	**Both sarcopenia and high Cardiac Metabolic Index**	**1.61 (0.90,2.87)**	**2.33 (0.69,7.83)**
	Diabetes		
	**Neither sarcopenia or high Cardiac Metabolic Index**	**Ref.**	**Ref.**
	**High Cardiac Metabolic Index only**	**1.81 (0.71,4.60)**	**1.36 (0.20,9.26)**
	**Sarcopenia only**	**1.24 (0.49,3.14)**	**1.90 (0.21,17.46)**
	**Both sarcopenia and high Cardiac Metabolic Index**	2.76 (1.14,6.71)*	10.49 (1.30,84.82)*
	Stroke		
	**Neither sarcopenia or high Cardiac Metabolic Index**	**Ref.**	**Ref.**
	**High Cardiac Metabolic Index only**	**2.13 (0.80,5.68)**	**1.33 (0.28,6.29)**
	**Sarcopenia only**	**1.97 (0.77,5.02)**	**1.44 (0.28,7.41)**
	**Both sarcopenia and high Cardiac Metabolic Index**	3.21 (1.26,8.20)*	**1.60 (0.29,8.91)**
	Hypertension		
	**Neither sarcopenia or high Cardiac Metabolic Index**	**Ref.**	**Ref.**
	**High Cardiac Metabolic Index only**	1.48 (0.99,2.18)*	**0.74 (0.35,1.56)**
	**Sarcopenia only**	**1.13 (0.78,1.65)**	**0.98 (0.49,1.97)**
	**Both sarcopenia and high Cardiac Metabolic Index**	**1.43 (0.96,2.13)**	**1.30 (0.60,2.81)**
	CMM		
	**Neither sarcopenia or high Cardiac Metabolic Index**	**Ref.**	**Ref.**
	**High Cardiac Metabolic Index only**	3.45 (1.47,8.07)**	**1.84 (0.47,7.20)**
	**Sarcopenia only**	2.88 (1.26,6.62)*	4.83 (1.22,19.06)*
	**Both sarcopenia and high Cardiac Metabolic Index**	4.82 (2.10,11.07)***	7.31 (1.72,31.15)**

HR, hazard ratio; CMM, Cardiometabolic multimorbidity; CMI, cardiac metabolic index;

Model 1: unadjusted;

Model 2: adjusted age, sex, residence, education, marital status, smoking, drinking, BMI, SBP, DBP, strenuous exercise, moderate intensity exercise, White Blood cell, Hemoglobin, Platelets, CRP, Total Cholesterol, LDL-c, BUN, Creatinine, Glucose, Uric Acid. * indicates P<0.05, ** indicates P<0.01, *** indicates P<0.001.

## Discussion

This study examines the relationship between sarcopenia, CMI, and CMM in 4959 middle-aged and older adults. The findings indicate that sarcopenia combined with high CMI is associated with an increased risk of CMM development, particularly among individuals aged 65 years or older. However, neither sarcopenia nor high CMI alone significantly elevates the risk of CMM. These findings suggest that the coexistence of sarcopenia and high CMI may increase the risk of CMM development.

The study also found a prevalence of possible sarcopenia, sarcopenia, and severe sarcopenia at 26%, 8.8%, and 3% respectively. These rates were notably lower than those reported by Wu et al. (38.5%, 18.6%, and 8%). This discrepancy may be related to the differences in the age and years of age of the participants (26). According to the AWGS criteria, the prevalence of sarcopenia in Asian countries ranges from 5.5% to 25.7%, which is consistent with our findings (13). Previous epidemiological studies have shown that sarcopenia is associated with the risk of several metabolic diseases, including insulin resistance, which is one of the main pathological mechanisms leading to CMM^6^ (27),. Skeletal muscle is a major organ for insulin action, and decreased skeletal muscle mass affects insulin-mediated glucose metabolism causing insulin resistance. A recent GWAS study conducted in a European population discovered that insulin resistance mediates the causal link between sarcopenia-related phenotypic grip strength and whole-body muscle mass with five distinct types of chronic metabolic diseases, including type 2 diabetes mellitus, nonalcoholic fatty liver disease, hypertension, coronary heart disease, and myocardial infarction (28). Furthermore, Ke et al. reported that sarcopenia-related phenotypic grip strength independently predicts the morbidity of chronic metabolic diseases and all-cause mortality (29). Similarly, our study found that individuals aged 65 years or older with sarcopenia exhibited a higher propensity for developing CMM compared to those without sarcopenia, aligning with previous findings. However, there was no significant increase in the prevalence of CMM among individuals with sarcopenia who were under 65 years of age compared to participants without sarcopenia. This lack of increase may be due to the small sample size included and the short follow-up period. Therefore, assessment of sarcopenia during community health screening may help identify patients with CMM and is expected to develop early interventions to improve health outcomes, especially in the older adult.

This study found that a high level of CMI increases the risk of CMD, consistent with previous cross-sectional studies (21). In the same cohort study, Zhu et al. suggested that high levels of BRI were associated with a high risk of CVD (30). The BRI index is a measure of abdominal obesity that assesses body fat distribution using waist and hip circumference but cannot distinguish between visceral and subcutaneous fat. It is crucial to consider this when evaluating the health risks associated with obesity. It is important to note that the distribution of fat in the body has a greater impact on human metabolism than total body fat. Visceral obesity, which is characterized by the accumulation of visceral fat, has been identified as a strong predictor of CMM morbidity and mortality (31). WHtR is considered a valuable parameter that reflects both subcutaneous adipose tissue (SAT) and visceral adipose tissue distribution. TG/HDL-C is an atherogenic marker that represents insulin resistance and cardio-metabolic risk. It offers stronger predictive value for stroke, CVD, and all-cause mortality compared to traditional lipid profiles (32). Therefore, CMI may be an important predictor of metabolic disease and is significantly associated with cumulative cardiometabolic risk factors (33, 34). The longitudinal analysis showed that the incidence of CMM was 8.7/1000 person-years in the group with neither sarcopenia nor high CMI, 17.37/1000 person-years in the high CMI group, 14.22/1000 person-years in the sarcopenia group, and 22.34/1000 person-years in the group with sarcopenia combined with high CMI. These data strongly demonstrate that sarcopenia combined with high CMI is a significant predictor of CMM incidence.

Previous extensive research has demonstrated that the loss of skeletal muscle mass and function is often accompanied by a relative or absolute increase in body fat, a condition known as sarcopenic obesity (SO) (35). So is associated with a higher risk of metabolic and cardiovascular diseases compared to sarcopenia or obesity alone. In a prospective study of 3,366 older adults living in a community, the highest risk of CVD events was associated with SO, assessed using waist circumference and muscle strength (36). Zhou et al. assessed the impact of SO on patients with type 2 diabetes using different measures of obesity. They found that when categorized using percent body fat (BF%), SO was associated with increased cardiometabolic risk in patients. Hye et al. reported that weight-adjusted waist-to-height ratio (WWI) was the most suitable predictor of cardiovascular events in older adults with T2DM and sarcopenia (37). Our study findings revealed that, after adjusting for all relevant variables, individuals with sarcopenia combined with CMI exhibited a significantly increased risk of CMM morbidity compared to those with either condition alone. This risk was particularly pronounced in those aged over 65 years and thus underscores the importance of assessing sarcopenia and CMI in community health screenings and routine clinical practice. It helps identify individuals at a higher risk of developing CMM and enables early intervention measures to minimize its occurrence.

Although our study has yielded several noteworthy observations, there are still some limitations. First, the exclusion of participants with incomplete sarcopenia information and missing CMD data during follow-up may have introduced selection bias, limiting the generalizability of our findings. Second, while we employed equations to estimate muscle mass, validated in Chinese individuals, potential differences in accuracy compared to the AWGS-recommended DAX should be noted;Third, despite adjusting for numerous conventional confounders, residual confounding by unaccounted factors such as body fat mass and medication history may have influenced our results. Four, we acknowledge that our decision to group sarcopenia and severe sarcopenia may impact the interpretation of our results. This approach was taken to maintain statistical power and to align with the clinical focus on the broader implications of muscle wasting on health outcomes. While this may obscure some differences between the two groups, it also provides a more generalizable assessment of the impact of muscle wasting across the spectrum of severity. Future research with larger sample sizes may benefit from distinguishing between sarcopenia and severe sarcopenia to better understand the nuances of their respective contributions to cardiometabolic health. Last, possible excessive use and the reliance on self-reported physician diagnoses for CMM may have introduced information bias. Additionally, our study has chosen to focus on individuals with high CMI, we recognize the potential value in examining the lower end of the CMI spectrum. However, given the specific objectives of this research to investigate the correlation between high CMI and cardiometabolic multimorbidity, we deliberately concentrated our efforts on the group with the greatest clinical relevance. Future studies may consider exploring the implications of low CMI in depth, which could provide a complementary perspective to our findings. Despite these limitations, our study provides valuable insights into the impact of sarcopenia and visceral obesity on cardiometabolic diseases, offering crucial clues for future research. Therefore, the assessment of sarcopenia and CMI should be clinically incorporated into community health screenings and routine clinical practice for older adults.

## Conclusion

In summary, the findings suggest a significant correlation between sarcopenia and CMI with CMM in middle-aged and older adults in China. The risk of CMM was significantly higher for sarcopenia combined with high CMI than for either condition alone. It is crucial to emphasize that preventing sarcopenia and/or implementing effective interventions for reducing CMI may contribute to reducing the incidence of CMM and promoting healthy aging.

## Data availability statement

The datasets presented in this study can be found in online repositories. The names of the repository/repositories and accession number(s) can be found below: https://charls.pku.edu.cn.

## Ethics statement

The studies involving humans were approved by institutional and national research council ethical. The studies were conducted in accordance with the local legislation and institutional requirements. The participants provided their written informed consent to participate in this study.

## Author contributions

LH: Funding acquisition, Writing – original draft. CL: Formal analysis, Visualization, Writing – original draft. YT: Methodology, Writing – original draft. YY: Data curation, Investigation, Writing – original draft. ML: Formal analysis, Writing – original draft. HT: Writing – review & editing. JL: Writing – review & editing.
